# Type 2 immunity in the brain and brain borders

**DOI:** 10.1038/s41423-023-01043-8

**Published:** 2023-07-10

**Authors:** Tornike Mamuladze, Jonathan Kipnis

**Affiliations:** 1 Center for Brain Immunology and Glia (BIG), Washington University in St. Louis, St. Louis, MO 63110 USA; 2 Department of Pathology and Immunology, School of Medicine, Washington University in St. Louis, St. Louis, MO 63110 USA; 3 Immunology Graduate Program, School of Medicine, Washington University in St. Louis, St. Louis, MO 63110 USA

**Keywords:** Type 2 immunity, Meningeal immunity, Neuroimmunology, Neuroimmunology, Interleukins

## Abstract

Recent research in neuroimmunology has revolutionized our understanding of the intricate interactions between the immune system and the central nervous system (CNS). The CNS, an “immune-privileged organ”, is now known to be intimately connected to the immune system through different cell types and cytokines. While type 2 immune responses have traditionally been associated with allergy and parasitic infections, emerging evidence suggests that these responses also play a crucial role in CNS homeostasis and disease pathogenesis. Type 2 immunity encompasses a delicate interplay among stroma, Th2 cells, innate lymphoid type 2 cells (ILC2s), mast cells, basophils, and the cytokines interleukin (IL)-4, IL-5, IL-13, IL-25, TSLP and IL-33. In this review, we discuss the beneficial and detrimental roles of type 2 immune cells and cytokines in CNS injury and homeostasis, cognition, and diseases such as tumors, Alzheimer’s disease and multiple sclerosis.

## Introduction

**Fig. 1:**
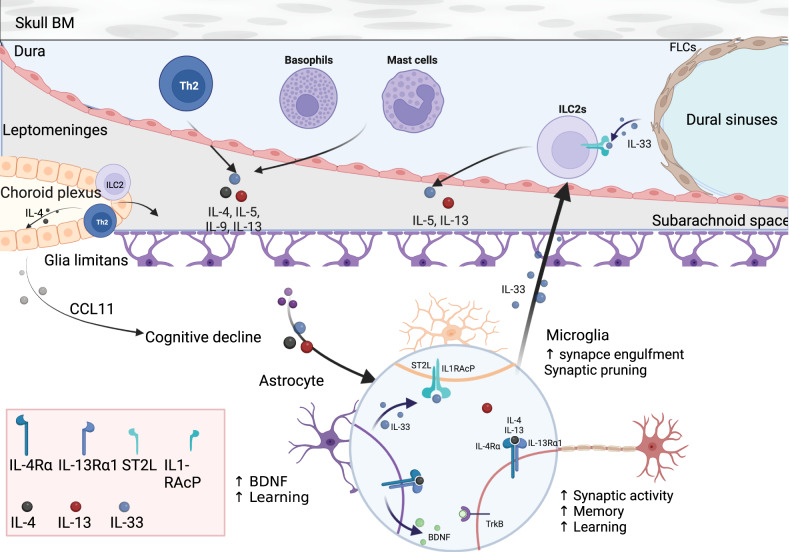
Neuroimmune circuitry in the brain and borders. Cytokines secreted by mast cells, ILC2s, and Th2s located in the meninges and choroid plexus modulate behavior and learning through receptors expressed by neurons and glia cells. Specifically, IL-4 can mediate its effect directly through the IL-4Rα expressed on GABAergic neurons, and astrocytes, in response to IL-4, produce brain-derived neurotropic factor (BDNF), a key molecule in learning and memory. Th2 cells accumulate in the choroid plexus, resulting in a local excess of IL-4 that acts on choroid plexus epithelial cells to produce CCL11, which has been correlated with the cognitive decline observed during aging. In addition, IL-13-deficient mice exhibit cognitive impairment similar to that observed in IL-4 knockout (KO) mice. IL-13 was also shown to be a synaptic protein in mouse and human brains, localized in the presynaptic membrane, whereas IL-13Rα1 is localized on the postsynaptic membrane. The engagement of IL-13 with type 2 receptors results in the phosphorylation of the NMDAR and AMPAR subunits and increases synaptic activity

The mammalian central nervous system (CNS) has only a limited capacity for self-repair and renewal. Inflammation within the CNS can be detrimental to neurons. Therefore, the presence of immune cells in the CNS was long considered a hallmark of pathology. Furthermore, the absence of lymphatic vessels inside the brain parenchyma [1], the low to no expression of MHC II by brain-resident microglial cells during homeostasis, the absence of other antigen-presenting cells, and the presence of a blood−brain barrier that prevents immune-cell recruitment inside the brain led to and supported the notion that the brain is an “immunologically privileged” organ [2, 3]. Recent advances in the understanding of neuroimmune interactions in the brain and its borders have shifted our understanding of the role of the peripheral immune system in CNS homeostasis and disease [4, 5] (Fig. 1).

Meninges, the three-layer protective membranes surrounding the brain and spinal cord, were traditionally considered passive barriers, but it is now clear that they are actively involved in regulating immune responses in the CNS. Single-cell profiling of meninges has revealed that both the leptomeninges (arachnoid mater and pia mater) and the dura mater are immune-rich tissues harboring cells of diverse lymphoid and myeloid lineages. Cerebrospinal fluid (CSF) from the brain can directly access the cranial meninges, where CNS-derived antigens are continuously sampled by dural sinus-associated antigen-presenting cells [6]. Moreover, within the meningeal dural layer, a functional lymphatic system drains antigens and macromolecules from the CNS to be sampled by the draining cervical lymph nodes [7, 8]. These findings have led to the understanding that within the meninges, the CNS harbors compartmentalized immune niches containing a rich repertoire of immune cells [9–12] that affect CNS homeostasis and pathologies [13, 14]. Elegant studies of novel mouse models have revealed that inflammation, in the context of sterile injury, is beneficial to the damaged CNS [15, 16]. Recent advances in the anatomical, molecular and cellular composition of the brain borders are reviewed elsewhere [4].

We now know that inflammation and the immune system are involved in almost all types of human acute and chronic diseases, including mental and physical health problems [17], and that they play a major role in enforcing homeostasis, as well as in the functional and structural integrity of the tissues, including the brain [18]. Homeostasis and inflammation are fundamentally connected, and most pathological states have a homeostatic counterpart [19]. In the spectrum of inflammatory responses, physiological inflammation occurs in the absence of infection or injury and is aimed at restoring the system to a homeostatic state. When this response is insufficient, type 2 inflammation-associated signals such as cytokines, chemokines, bioactive amines, and immune cells play a role in acute and chronic inflammation [20].

Type 2 response-associated immune cells, including T helper 2 (Th2) cells, innate lymphoid type 2 cells (ILC2), eosinophils, mast cells, basophils and alternatively activated macrophages, are associated with either a protective or a pathogenic phenotype depending on the circumstances [21]. Originally, it was thought that the primary role of type 2 immunity was to limit the consequences of type 1-driven inflammation. While it is true that type 1 inflammation caused by structural loss is often followed by a type 2 inflammatory response, it is also often initiated owing to a loss of function [20] caused by noxious substances such as allergens [22], xenobiotics, and parasitic worms [23] that can disrupt epithelial barriers [24]. Inflammation caused by structural loss (type 1), functional loss (type 2), and regulatory loss (type 3) can antagonize other types of responses. In some cases, the induction of type 2 immunity limits the inflammation caused by a Th1/Th17 response and is beneficial for the host. In the case of experimental autoimmune encephalomyelitis *(*EAE), interleukin-4 (IL-4)-induced type 2 immunity suppresses type 1 driven inflammation, thereby ameliorating EAE [25]. On the other hand, it is crucial for the host to mount appropriate immune responses against invading pathogens. For example, Leishmania major requires a Th1 response to clear the infection; therefore, in BALB/c mice, where CD4+ T cells differentiate into Th2 cells in response to injury, these cells are unable to control the infection, a result that is detrimental to the host. Moreover, pathogenic activation of type 2 immunity in response to harmful environmental stimuli can contribute to the development of diseases/conditions that include asthma, allergic rhinitis, atopic dermatitis, and allergies to drugs and foods [26], while excessive inhibition of cytotoxic type 1 immunity by IL-4- and IL-13-activated macrophages can promote tumor development as well as pathological fibrosis or organ scarring [27]. It is therefore crucial for the CNS to tightly regulate the induction of inflammation or to limit its pathogenic activation.

## Cytokines involved in the initiation of the type 2 response

IL-33, IL-25 and thymic stromal lymphopoietin (TSLP) are crucial cytokines that play a central role in the induction of type 2 inflammation. IL-33 belongs to the IL-1 family of cytokines and is released as an alarmin in response to cell injury or tissue damage. IL-33 acts on cells expressing the ST2 receptor, also known as interleukin 1 receptor-like 1 (IL1RL1) [28], including mast cells, ILC2s, Tregs, Th2 cells, eosinophils, basophils, dendritic cells (DCs), and macrophages (including CNS-resident microglia) [29]. IL-33 has a multifaceted role in homeostasis and pathology and is expressed by many cell types, including hematopoietic, stromal, and parenchymal cells in the CNS and its borders [6, 30].

IL-33 is highly expressed in developing brain astrocytes and oligodendrocytes [31, 32]. Astrocytes residing near redundant synapses release IL-33 to recruit microglia, promoting synapse engulfment by microglia. A disruption of the IL-33 axis leads to overactive brain circuitry, behavioral abnormalities and seizures owing to increased numbers of excitatory synapses [33], presumably because of microglial dystrophy. Moreover, IL-33 is upregulated in the mature brain by astrocytes upon Toll-like receptor (TLR) stimulation [34] and is constitutively expressed by oligodendrocytes [35]) and neurons [36]. The IL-33/ST2 axis has been implicated in many CNS-related diseases and conditions, including Alzheimer’s disease [37, 38], age-related macular degeneration [39], multiple sclerosis (MS) [40] and EAE [41–43], stroke [44], CNS injury [35, 45, 46], and pain [47].

Findings from studies in mice suggest that IL-33 is protective in models of stroke [44] and CNS injury [35]. In the healthy brain, IL-33 is highly expressed by oligodendrocytes. Upon injury, IL-33 is released through an unknown mechanism and promotes monocyte recruitment to the injured site [35]. Mice lacking IL-33 have impaired recovery following spinal cord injury (SCI). Upon its release, IL-33 can also activate ILC2s residing in the meninges, which in turn upregulate IL-5 and IL-13, inducing type 2 immunity [46].

MS patients have elevated IL-33 levels in the brain and plasma [40]. This increase in IL-33 might be a compensatory mechanism toward Th1/Th17-mediated inflammation. ST2 expression increases in the spinal cord after EAE, and ST2-deficient mice have exacerbated EAE [43]. IL-33 treatment can reduce IL-17 and IFN-y levels, most likely by inducing IL-5 and IL-13 [43]. Another possible role of the IL-33/ST2 axis in EAE is mediated through CNS regulatory T cells (Tregs), which also express ST2. The deletion of ST2 in Foxp3+ Tregs leads to EAE exacerbation by diminishing the ability of Tregs to suppress IL-17A-secreting gamma-delta T cells [48]. IL-33 function can be modulated via cleavage by proteases and tryptases secreted by neutrophils and by activated mast cells. Cleaved IL-33 is a potent activator of ILC2s and eosinophils [49]. Importantly, mast-cell-derived IL-33 activates ILC2s in the meninges, which in turn promote a Th2 response in the SJL mouse model of MS, limiting the progression of the disease [42].

IL-33 function in chronic neurodegenerative diseases is less well understood. The first evidence comes from human patients. IL-33 is decreased in the brains of patients with Alzheimer’s disease. Moreover, IL-33 polymorphisms modulate the risk of Alzheimer’s disease, with some rare single-nucleotide polymorphisms (SNPs) found to be protective [37]. Soluble ST2, a decoy receptor for IL-33, is upregulated in the serum of patients with mild cognitive impairment. Intraperitoneal administration of IL-33 enhances microglial phagocytosis and improves synaptic impairment and Aβ aggregation in APP/PS1 mice [38].

In conclusion, IL-33 plays a crucial role in inflammation and homeostasis, and its multifaceted role is still being researched. The involvement of IL-33 in CNS-related diseases, including Alzheimer’s disease, MS and EAE, highlights its potential as a therapeutic target. Further work is needed to better understand the mechanisms underlying the role of IL-33 and to identify its potential as a therapeutic agent.

The functions of other type 2 immunity inducers in the CNS are less well characterized. IL-25, for example, may be involved in EAE and MS: its expression in microglia is increased after EAE induction, and it is important in maintaining the blood−brain barrier and limiting Th17-mediated inflammation. Accordingly, IL-25-deficient mice are highly susceptible to EAE [50]. The protective role of IL-25 is also mediated through the upregulation of IL-13 by Th2 cells and potentially by ILC2s, which inhibit IL-23, IL-1b, and IL-6 expression in DCs. Furthermore, human brain capillaries express IL-25, which is downregulated in tissues near severe MS lesions [51].

## Th2 cells and effector cytokines

IL-4 and IL-13 were among the first cytokines identified, and their functions often overlap. IL-4 can elicit its function by binding with two types of receptors: the type 1 receptor, which is expressed mainly on hematopoietic cells and uses IL-4Rα and the common gamma chain, and the type 2 receptor, which is expressed on nonhematopoietic cells and possesses two subunits, IL-4Rα and IL-13Rα1. The cytokine IL-13, on the other hand, can bind only with the type 2 receptor. The binding of IL-4 with the type 1 complex phosphorylates JAK1/3, which in turn phosphorylates tyrosines within IL-4Rα cytoplasmic domains, creating docking sites for STAT6 and insulin receptor substrate 2 (IRS2). In naïve CD4+ T cells, STAT6 induces the expression of GATA3 and thus leads to their differentiation into Th2 cells. In B cells, STAT6 induces class switching to IgE and promotes alternative activation of macrophages [52]. The type 2 receptor is associated with JAK1 and TYK2, leading to STAT6 phosphorylation, homodimerization, and nuclear translocation [53]. IL-13, in addition to binding with the type 2 receptor, binds with the decoy receptor IL-13Rα2, which lacks a cytoplasmic domain and therefore has a higher affinity for IL-13. IL-13Rα2 is often overexpressed in malignant tumors, especially in glioblastoma multiforme (GBM). Newer findings suggest that in addition to IL-13 signaling, IL-13Rα2 can attenuate IL-4 signaling by inhibiting the dimerization of IL-4Rα with second subunits. Furthermore, IL-13Rα2 promotes transforming growth factor beta (TGF-b) production in monocytes and thus promotes tissue fibrosis [54]. IL-4 and IL-13 are important in reducing IL-1-induced inflammation by increasing the expression of IL-1R2, a decoy receptor for IL-1β that limits inflammation [55].

Recent research has revealed roles of IL-4 and IL-13 in the CNS beyond their established functions in immune regulation and inflammation. T cells have long been implicated in the modulation of learning and behavior [16, 56]. In the healthy adult CNS, T cells reside mainly in the meninges and are not found in the brain parenchyma [6]. IL-4 and IL-13 produced by meningeal T cells are involved in learning behaviors [57, 58]. IL-4-deficient mice were shown to exhibit learning deficits [58]. Similarly, SCID and CD4-depleted mice were found to exhibit impaired learning in different behavioral tasks. IL-4Rα is expressed in some brain-resident cells, including neurons. Earlier studies suggested that IL-4 mediates its effect on learning through astrocytic IL-4Rα [58–60], whereby astrocytes, in response to IL-4, produce brain-derived neurotropic factor (BDNF), a key molecule in learning and memory. A new line of evidence suggests that cytokines can directly modulate neuronal responses through receptors expressed on neurons [61–63]. Similarly, a recent study showed that IL-4 can mediate its effect directly through IL-4Rα expressed on GABAergic neurons. Conditional ablation of IL-4Rα on GABAergic neurons but not microglia or macrophages resulted in impaired memory. Moreover, memory deficits were restored after the injection of wild-type CD4 cells into SCID mice but not if transferred T cells were IL-4 deficient [64].

Resident T cells in the choroid plexus are another source of IL-4 in the brain [65]. As mice age, Th2 cells accumulate in the choroid plexus, resulting in a local excess of IL-4 that acts on choroid plexus epithelial cells to produce CCL11 [65]. This increase in CCL11 has been correlated with the cognitive decline observed during aging [66] and has also been implicated in the cognitive syndrome observed in patients with long COVID-19 [67].

In addition to IL-4, other Th2-derived cytokines have also been implicated in the modulation of behavior and learning. IL-5 was shown to enhance cognitive function, specifically spatial recognition, in mouse models of Alzheimer’s disease [68].

IL-13-deficient mice exhibit cognitive impairment similar to that observed in IL-4 knockout (KO) mice [57]. While potential sources of IL-4 in the CNS include mostly immune cells, such as Th2 cells, mast cells and basophils, IL-13 was also shown to be a synaptic protein in mouse and human brains. This cytokine is localized in the presynaptic membrane, whereas IL-13Rα1 is localized on the postsynaptic membrane. The engagement of IL-13 with type 2 receptors results in the phosphorylation of the NMDAR and AMPAR subunits and increases synaptic activity [69].

In addition to modulating mammalian behavior, type 2 cytokines have been implicated in CNS diseases and conditions, including depression [70], injury [15], Alzheimer’s disease [71], MS, stroke [72], and traumatic brain injury [69, 73]. IL-13 expression is enhanced during traumatic brain injury in human neurons, probably as a compensatory protective mechanism in the case of excitatory death [69]. The neuroprotective role of CNS-infiltrating Th2 cells during SCI or optic nerve crush is independent of the binding of the T-cell receptor (TCR) with its cognate antigens [15]. Th2 cells promote functional recovery after spinal cord and optic nerve injury [15]. The protective effect of IL-4 has also been demonstrated in a mouse model of Alzheimer’s disease, where the injection of IL-4 alone or of IL-4 + IL-13 induced the proliferation of Arg1+ microglia and the clearance of plaques in APP/PS1 mice [71, 74]. Other studies, however, have found contraindicatory results [75].

The protective role of type 2 immunity in Th1/Th17-mediated diseases is supported by studies using knockout mouse models. For example, mice lacking IL-10 are more susceptible to the development of severe EAE than wild-type mice, indicating that IL-10 plays a critical role in regulating the immune response in this disease. In contrast, IL-4 KO mice showed no such effect [76, 77]. This may be due to the different polarizing effects of IL-4 and IL-10 [78]. IL-4 induces macrophage polarization through STAT6, whereas IL-10 acts through STAT3 signaling. Interestingly, IL-10-activated microglia closely resemble human microglia in Nasu–Hakola disease, which is caused by mutations in the TREM2 or DAP12 genes [79].

## Mast cells

Mast cells have long been considered master regulators of allergic inflammation. We are now also beginning to recognize their role in homeostasis [80] and in responses such as the recruitment of neutrophils during skin inflammation [81], host defense against bacteria [82], inflammatory pain [83], and immunosuppression [84]. Mast cells in the healthy CNS reside in the meninges, mostly in the dura, and presumably also in the brain parenchyma in small numbers [85–87]. Originating from late erythro-myeloid progenitors [88], mast cells mature in tissues and acquire unique gene signatures in different niches [89]. In addition to their expression of the canonical mast-cell tryptase and proteases, mast cells express high levels of IL-4 and IL-13. They also exhibit immunoregulatory functions, and after IL-33 stimulation, they can suppress allergic inflammation by secreting IL-2, which in turn promotes Treg expansion [90], thus making them prime candidates for mounting type 2 responses [91].

In the brain, mast cells were first identified in 1890, near MS brain lesions [92]. Their involvement in the pathogenesis of MS is further supported by human studies. Mast cell-specific tryptase is elevated in the CSF of MS patients [93]. Their role in the pathogenesis of MS, however, is still not fully understood [94]. In histochemical studies, brain mast cells seemed to lack expression of c-Kit and FCER1A, canonical markers of tissue mast cells [95, 96], making the study of brain-resident mast-cell function particularly challenging.

Early studies on mast-cell involvement in the pathogenesis of EAE development, carried out largely on W/Wv and Kit^W-sh/W-sh^ mouse models, showed that mast-cell-deficient W/Wv mice demonstrated delayed onset and reduced severity of EAE [97]. The transplantation of bone marrow-derived mast cells restored the clinical course of this disease. Notably, transplantation failed to reconstitute brain mast cells, suggesting that peripheral mast cells might play a role in such restoration [98]. In contrast, Kit^w-sh^ mice demonstrated exacerbated EAE disease, and peripheral restoration of mast cells via bone marrow-derived mast-cell transplantation was insufficient to change its clinical course, indicating a complex role for mast cells in EAE [98]. Extensive studies have aimed to elucidate the role of meningeal mast cells in the efficient infiltration of inflammatory T cells into the CNS during EAE development. Mast cells in the meninges are strategically located near the meningeal vasculature and dural sinuses. They promote CNS recruitment of immune cells by secreting proinflammatory mediators such as tumor necrosis factor, which can activate nearby endothelial cells and enhance leukocyte adhesion and extravasation into the CNS [99]. Meningeal mast cells in EAE mice have been shown to play a crucial role in the efficient CNS infiltration of inflammatory T cells. The transfer of mast cells into W/Wv mice was shown to reconstitute meningeal mast cells, which alleviated EAE severity and delayed EAE onset, thus highlighting the importance of these cells in EAE pathogenesis. Meningeal mast cells also recruit neutrophils to the site of inflammation, which can further exacerbate tissue damage in the CNS [99].

Mast cells have been found to play critical roles in CNS homeostasis, including the modulation of mammalian behavior. Given their identification as immune cells containing heparin and histamine, mammalian mast cells are morphologically consistent with the mast cells observed in ancient organisms such as tunicates and crustaceans, suggesting that these cells coevolved with the nervous system. Mast cell function is tightly regulated by neurotransmitters and neuropeptides such as acetylcholine, gamma-aminobutyric acid (GABA), glutamate, dopamine, substance P, vasoactive intestinal peptide (VIP), and calcitonin gene-related peptide (CGRP) [100]. CGRP-mediated mast-cell activation is particularly important in CNS homeostasis; for example, neurogenic inflammation leading to migraine is mediated through CGRP. Moreover, chemical degranulation of meningeal mast cells with Compound 48/80 leads to prolonged excitation of nociceptors in the meninges, with downstream trigeminal activation [101]. This neuroimmune communication seems to be bidirectional. In Kit^w-sh^ mice, mast-cell deficiency has been associated with behavioral abnormalities and an anxiety-like phenotype. In addition, pharmacological inhibition of mast-cell degranulation by intracerebroventricular injection of disodium cromoglycate increased anxiety-like behavior in mice. No significant effect was observed after intraperitoneal injection, suggesting the importance of mast cells residing in the CNS [102].

New evidence suggests that mast cells may actually be protective under certain conditions. Studies in Kit^w-sh^ mast-cell-deficient mice have shown that mast cells may be protective in models of SCI and mechanical brain injury [103, 104]. Compared to wild-type controls, Kit^w-sh^ mice exhibited significantly higher T-cell infiltration and reduced functional recovery in these experiments. It was suggested that these effects are mediated through MCP4 cleavage of MCP-1, IL-6 and IL-13, and mice lacking mMCP4 showed defects in functional recovery after SCI [104].

Despite the findings discussed above, there is a need for critical reevaluation, as the lack of mast-cell-specific mouse models has contributed to ambiguity in the field. The first-generation mast-cell-deficient mouse model, W/Wv, has severe hematopoietic perturbations, including anemia, as well as reduced γδ T cells, basophils, and neutropenia. Similarly, Kit^W-sh/W-sh^ mice demonstrate enhanced myelopoiesis and a subsequent increase in neutrophils and basophils. Studies with new mast-cell-deficient models that are not based on those first-generation models have failed to replicate many findings attributed to mast cells, including their role in EAE [105]. In the CPA3 ^-/-^ mouse model, which lacks connective tissue mast cells but also has reduced numbers of basophils in the blood, researchers found no involvement of mast cells in autoimmune diseases and failed to reproduce differences in EAE susceptibility in W/Wv mice [106].

## ILC2s

Recent findings on ILC2s have shed light on their diverse functions in various biological processes. ILC2s are highly enriched in barrier surfaces, where they play crucial roles in initiating type 2 immune responses in adipose tissue homeostasis [107], lung homeostasis and inflammation [108, 109], and gut barrier functions [110].

The majority of tissue-resident ILC2s in adults are generated de novo during the postnatal period. Owing to their transcriptional and functional similarities, ILC2s were initially considered to be an innate counterpart of Th2 cells. However, other subtypes of ILC2s have since been described, such as the IL-10-producing killer cell lectin-like receptor G1 (KLRG1)+ ILC2s, which suppress Th2 responses and induce tolerance [111], and type 2 induced inflammatory ILC2s (iILC2s), which are recruited to the lungs from the intestines in response to exposure to IL-25 or to parasitic worms [112]. ILC2s are activated by IL-33, IL-25, and TSLP in the lungs, gut, and skin [113–115]. Moreover, skin ILC2s express IL-4Ra and can be activated by basophil-derived IL-4 in inflamed mouse and human skin [116] as well as in the inflamed lung, where activated ILC2s promote eosinophil infiltration through the secretion of IL-5 and IL-13 [117].

In addition to their roles in type 2 immune responses and tissue homeostasis, ILC2s play a role in hematopoiesis. ILC2s are present in bone marrow, including skull bone marrow, and can stimulate hematopoiesis. In the context of 5-fluorouracil (5-FU)-induced stress, B-cell progenitor-derived IL-33 activates myeloid differentiation primary response 88 (MyD88)-mediated secretion of granulocyte-macrophage colony-stimulating factor (GM-CSF) in ILC2s to support myeloid hematopoiesis. Moreover, ILC2s can expand and become activated during stress, upregulating programmed cell death protein (PD)-1, ST2 and cluster of differentiation 25 (CD25) and downregulating IL-7Rα. In granulocyte-macrophage colony-stimulating factor (GM-CSF) KO mice, the transplantation of wild-type ILC2s was found to be sufficient to restore hematopoiesis [118]. IL-33 has also been shown in certain contexts to induce IL-10 expression and downregulate type 2 cytokine production in lung ILC2s [111].

ILC2 function is tightly regulated by the nervous system through the action of neurotransmitters. Both peptidergic and nonpeptidergic neurotransmitters, such as neuromedin U (NMU) and VIP, promote ILC2 function [119, 120]. Conversely, the engagement of B2 adrenergic receptors with epinephrine inhibits ILC2 proliferation and function [121]. During inflammation, this neuroimmune circuit becomes even more complicated, as other cells can affect receptor expression on ILC2s and further modulate their activation and function, such as basophils, which have been shown to affect ILC2 function through the regulation of neuromedin B expression on ILC2s during lung infection with the nematode *Nippostrongylus brasiliensis* [122].

The interplay between ILC2s and the nervous system is further exemplified by the role of CGRP in regulating ILC2 function. CGRP and its receptors are expressed by ILC2s in barrier tissues such as meninges and the lungs, where excess CGRP can dampen IL-33-induced inflammation and inhibit the type 2 cytokine production and proliferation of ILC2s during airway inflammation and parasitic infection [109, 123]. Similarly, in the context of meningeal bacterial inflammation, CGRP released from nociceptors dampens macrophage activation and recruitment of neutrophils and monocytes [124]. Interestingly, nociceptor ablation in the meninges results in increased neutrophil and monocyte recruitment and protects the brain from invading pathogens. Furthermore, in the context of type 2 immunity, IL-5 produced by activated ILC2s and Th2 cells can activate nociceptors through IL-5R and induce the release of VIP, creating an inflammatory circuit loop that promotes allergic inflammation.

ILC2s were also shown to be present in the spinal dura and choroid plexus, albeit to a lesser extent than in the cranial meninges. Interestingly, no ILC2s were found in the brain parenchyma or perivascular space [46]. In the dura, ILC2s are mainly located near the dural sinuses, where they are in close proximity to IL-33-expressing fibroblast-like cells, similar to the situation observed in lung and white adipose tissue [125]. During lung infection, ILC2- and Th2-derived IL-13 promotes the expansion of these stromal cells, which in turn upregulate IL-33, creating a positive feedback loop [125].

Meningeal ILC2s express Thy1.2, ST2, CD25, spinocerebellar ataxia type 1 (Sca10), and low levels of c-Kit and IL-7Rα and are major producers of IL-13 in tissues. As tissue-resident cells, ILC2s exhibit tissue-specific receptor expression patterns, which are imprinted by local signals [126]. Likewise, dural ILC2s are distinct from lung ILC2s. The basal activation state of meningeal ILC2s is lower than that of lung-resident cells [46]. The proximity to brain-derived signals may explain the differences in basal activation observed between the two tissue types. ILC2 precursors originate from bone marrow. Future studies should determine the functional and phenotypic differences between blood-derived ILC2s and the adjacent bone marrow.

The role of ILC2s in neuroinflammation is an emerging field of study. ILC2 function is best characterized in the spinal cord injury model. After SCI, cranial meningeal ILC2s become activated in an IL-33-dependent manner, produce IL-5 and IL-13, and upregulate CGRP and its receptors. This effect is abolished in IL-33 KO mice. The reconstitution of IL-33 KO mice with wild-type ILC2s restores the protective effect of meningeal ILC2s, leading to better functional recovery and reduced lesion area [46]. Interestingly, ILC2s also accumulate in the injured spinal cord 10 days after injury, but the origins of these cells and their relative contributions from local meningeal bone marrow and circulating progenitors are unknown.

ILC2 functions are also linked to cognition. In a mouse model of Alzheimer’s disease (3xTg-AD mice), defects in meningeal ILC2s, including a reduction in cell number and functional deficits in IL-5 production, were identified [68]. During aging, ILC2s accumulate in the meninges and choroid plexus. ILC2s are practically absent in the choroid plexus of young mice. Aged choroid plexus ILC2s are quiescent at steady state, but upon stimulation, they produce IL-5 and IL-13. The transfer of activated choroid plexus ILC2s was shown to increase the cognitive performance of mice after 1 week [127].

A correlation has also been observed between EAE susceptibility and ILC2 numbers in the brain and meninges, with EAE-resistant mice having larger numbers [128]. Also found to have decreased numbers of ILC2s are W/Wv mice, which lack mast cells owing to the disruption of c-kit signaling. The relationship between ILC2s and mast cells in EAE pathogenesis requires further investigation.

## Tumors

Glioblastoma multiforme (GBM) is a highly aggressive brain tumor that can evade the surveillance mechanisms of the immune system, making it difficult to treat. The type 2 immune response is a complex mechanism that can be either beneficial or detrimental to cancer growth, depending on the context [129, 130]. On the one hand, the response can promote tumor dissemination and metastasis through the action of CD4+ Th2 effector cells, which regulate the pro-tumor properties of tumor-associated macrophages via IL-4 expression [131, 132], and IL-4 is upregulated in many mouse and human tumors [133]. Although their role in pathogenesis is still unknown, their enrichment in human and mouse GBM suggests that these effector cells may play a role in tumor growth [134]. Type 2 immunity is often regarded as an unfavorable antitumor response since it allows cancer cells to evade the immune system.

However, recent human epidemiological data suggest that the type 2 response can be an effective defense mechanism against cancer. For instance, there is an inverse correlation between allergy and the occurrence of glioblastoma (GBM) [135]. In addition, asthma-associated SNPs in IL-4R, IL-13, and STAT6 gene loci are inversely correlated with the occurrence of GBM [136, 137]. Eliciting a type 2 response in the early stages of different cancers can be protective [138, 139]. This effect can be mediated through various mechanisms, including the recruitment of eosinophils in an IL-5-dependent manner [140, 141], TSLP-mediated Th2 polarization [138], and IL-4-mediated reorganization of tumor vasculature with subsequent hypoxia and cancer cell death [142].

Another newly recognized type 2 antitumoral effect is mediated through IgE. IgE is an ancient immunoglobulin thought to have evolved to protect against infections caused by large parasites, such as helminths. Although IgE increases in abundance in the presence of helminth infections, it is not critical for protective immunity against these parasites, as it has been found to respond to environmental toxins and xenobiotics that may be carcinogens [143]. Early establishment of an IgE response via IL-4 toward carcinogenic DNA-damaging environmental xenobiotic 9,10-dimethylbenz-A-anthracene (DMBA) restricts tumor growth in a basophile FC epsilon receptor (FcεRIα)-dependent manner [144]. Furthermore, FcεRIα is associated with positive survival in the majority of human tumors, including GBM [145], and treatment with omalizumab, a monoclonal antibody against IgE, might be associated with a higher risk of developing cancer [146]. In addition, IL-13Rα2, a restrictive receptor for IL-13, is overexpressed in the majority of GBM patients, making it a prime candidate for chimeric antigen receptor (CAR) T-cell therapy [147]. These data indicate that GBM actively suppresses type 2 responses to promote tumor growth.

The manipulation of the meningeal lymphatic system has shown promise in enhancing immune responses against GBM. For example, ectopic expression of vascular endothelial growth factor (VEGF)-C enhances the meningeal lymphatic network and the drainage of tumor antigens into the deep cervical lymph nodes. The treatment of mice with VEGF-C was shown to result in the clearance of GBM cells, and this effect was dependent on both CD4 and CD8 T cells, as the depletion of these T cells abolished VEGF-C-driven protection [148].

GBM can further subvert immune activation by recruiting myeloid suppressor cells through the production of the chemoattractant CXCL12 [149]. The CXCL12 receptor CXCR4 is widely expressed on myeloid cells and is essential for their retention in bone marrow and for recruitment into the tissue. The disruption of the local CXCL12–CXCR4 axis in the bone marrow results in the rapid recruitment of monocytes and neutrophils into the meninges [150]. Local bone marrow in the skull and in vertebral columns are directly connected to the meninges via vascularized bone channels [151–153]. These adjacent bone marrow sites act as myeloid and lymphoid cell reservoirs in homeostasis, aging, and acute and chronic CNS pathologies such as stroke and EAE [150, 151, 154]. During stroke, skull bone marrow neutrophils are the primary responders in the initial stages of damage [151]. Moreover, in EAE, local bone marrow-derived monocytes have been shown, based on their gene signature, to be less inflammatory, raising the possibility of discrete functions of adjacent bone marrow and blood-derived cells [150]. Moreover, recent findings suggest that this route is bidirectional and that the brain can directly regulate the hematopoietic niche in adjacent bone marrow sites in mice as well in humans [155–157]. CSF from the brain can directly access adjacent bone marrow via these channels, an axis shown to be important in EAE and meningitis. Similarly, GBM-derived CXCL12 can aid in the recruitment of adjacent bone marrow-derived myeloid suppressor cells through those channels. These findings shed light on the complex interplay among the meningeal lymphatic system, the hematopoietic niche and GBM pathogenesis, suggesting new avenues for GBM immunotherapy.

## Conclusions and perspectives

Type 2 immunity is an adaptive immune response commonly associated with allergic inflammation or helminth parasite infection. It plays a crucial role in tissue repair and involves complex coordination between cells and cytokines. The induction of type 2 immunity in response to a loss of function aims to eliminate perturbators by inducing peristalsis, diarrhea, vomiting, itching, and sneezing and by complementing a basal function. Type 2 immunity also plays roles in glucose homeostasis [158], thermogenesis [159], and insulin resistance [160]. Mounting evidence suggests, moreover, that type 2 immune responses in the brain and brain borders could shape adaptive and dysfunctional neurological processes. Type 2 cytokines are crucial for maintaining CNS homeostasis. Two cytokines involved in the initiation of type 2 responses, IL-33 and IL-25, are highly enriched in the CNS. Moreover, effector cytokines such as IL-4 and IL-13 can be secreted by different cell types in the healthy CNS and act as neuromodulators by binding with cognate receptors expressed by CNS-resident cells, including neurons. The type 2 response also often limits the detrimental consequences of type 1/type 3 inflammation and aims to restore tissue homeostasis. Emerging evidence suggests that Th2 cells are beneficial for the damaged CNS. Dampening the type 2 response by knocking out cytokines, receptors or cells involved in the response often leads to an exacerbation of inflammation and pathology.

While in this review we have focused on the type 2 responses initiated at the brain borders or in the CNS parenchyma itself, growing evidence suggests that the peripheral tissue−CNS axis is implicated in many CNS disorders. In the case of the gut-brain axis, for example, different gut microbiota affect mammalian behavior and even the progression of neurodegenerative and autoimmune diseases [161]. Similarly, systemic inflammation induced by lipopolysaccharide (LPS) or IL-1b can modulate behavior [162]. However, less is known about how systemic type 2 pathological activation modulates CNS homeostasis and disease progression. People with asthma and other allergic conditions are less likely to develop GBM [137]. Some reports suggest a correlation between atopy and neurobehavioral conditions [163–166] and headaches [167, 168]. Moreover, there is a critical need to re-evaluate the conflicting data regarding the functions of immune cells involved in the type 2 response using better tools. Future studies should determine the extent to which type 2 immunity contributes to these effects and identify potential therapeutic targets.
